# Zinc Acquisition Mechanisms Differ between Environmental and Virulent Francisella Species

**DOI:** 10.1128/JB.00587-17

**Published:** 2018-01-24

**Authors:** G. Brett Moreau, Aiping Qin, Barbara J. Mann

**Affiliations:** a Department of Microbiology, Immunology, and Cancer Biology, University of Virginia, Charlottesville, Virginia, USA; b Department of Medicine, Division of Infectious Diseases and International Health, University of Virginia, Charlottesville, Virginia, USA; Rutgers University-Robert Wood Johnson Medical School

**Keywords:** Francisella novicida, Francisella tularensis, zinc, *znuABC*, *zupT*, *zur*

## Abstract

Zinc is an essential nutrient for bacterial growth. Because host cells can restrict pathogen access to zinc as an antimicrobial defense mechanism, intracellular pathogens such as Francisella must sense their environment and acquire zinc in response. In many bacteria, the conserved transcription factor Zur is a key regulator of zinc acquisition. To identify mechanisms of zinc uptake in Francisella novicida U112, transcriptome sequencing of wild-type and putative *zur* mutant bacteria was performed. Only three genes were confirmed as directly regulated by Zur and zinc limitation by quantitative reverse transcription-PCR. One of these genes, FTN_0879, is predicted to encode a protein with similarity to the *zupT* family of zinc transporters, which are not typically regulated by Zur. While a putative *znuACB* operon encoding a high-affinity zinc transporter was identified in U112, expression of this operon was not controlled by Zur or zinc concentration. Disruption of *zupT* but not *znuA* in U112 impaired growth under zinc limitation, suggesting that ZupT is the primary mechanism of zinc acquisition under these conditions. In the virulent Francisella tularensis subsp. tularensis Schu S4 strain, *zupT* is a pseudogene, and attempts to delete *znuA* were unsuccessful, suggesting that it is essential in this strain. A reverse TetR repression system was used to knock down the expression of *znuA* in Schu S4, revealing that *znuA* is required for growth under zinc limitation and contributes to intracellular growth within macrophages. Overall, this work identifies genes necessary for adaptation to zinc limitation and highlights nutritional differences between environmental and virulent Francisella strains.

**IMPORTANCE** Francisella tularensis is a tier 1 select agent with a high potential for lethality and no approved vaccine. A better understanding of Francisella virulence factors is required for the development of therapeutics. While acquisition of zinc has been shown to be required for the virulence of numerous intracellular pathogens, zinc uptake has not been characterized in Francisella. This work characterizes the Zur regulon in F. novicida and identifies two transporters that contribute to bacterial growth under zinc limitation. In addition, these data identify differences in mechanisms of zinc uptake and tolerance to zinc limitation between F. tularensis and F. novicida, highlighting the role of *znuA* in the growth of Schu S4 under zinc limitation.

## INTRODUCTION

Francisella tularensis is a highly infectious Gram-negative bacterium and the causative agent of the potentially life-threatening disease tularemia. Because of its low infectious dose, its ability to be easily aerosolized, and its potential lethality, this bacterium has been classified as a tier 1 select agent by the U.S. Centers for Disease Control and Prevention, signifying that it poses the greatest potential for lethality if deliberately misused. Because of the serious threat this organism poses to human health, its biology and virulence are critical areas of investigation.

Two of the most commonly used strains are Francisella novicida U112 and F. tularensis subsp. tularensis Schu S4. U112 is an environmental isolate, while Schu S4 was isolated from an infected patient (1). U112 is used as an experimental model of virulent F. tularensis infection because it has high genomic identity, can be manipulated under biosafety level 2 conditions, and although attenuated in humans, is still capable of causing disease in mice (2). An F. novicida transposon mutagenesis library with single transposon disruptions of all nonessential genes is available (3), allowing the identification of phenotypes of specific gene knockouts. However, there are differences in nutritional requirements and host responses between less virulent and more virulent Francisella strains (4–6), so findings on the less virulent F. novicida strains do not always apply to the F. tularensis subsp. tularensis strain. Differences in gene content are thought to be partially responsible for differences in virulence between these strains. Despite being 97% identical in gene content, F. tularensis subsp. tularensis Schu S4 has significantly fewer protein-encoding genes because of gene disruptions (1, 7). Many of these disruptions occur in metabolic pathways, making F. novicida less fastidious than F. tularensis subsp. tularensis (8).

Francisella is considered a facultative intracellular pathogen that resides primarily within host cells. After phagocytosis, Francisella escapes from the phagosome within about 1 h (9) and begins to replicate in the host cytosol at around 8 h postinfection (10). Intracellular replication is critical for virulence, as mutants that are unable to survive and replicate intracellularly are attenuated (11–13). The ability of the bacteria to scavenge nutrients from the host environment has proved vital to bacterial survival and replication. For example, F. tularensis subsp. tularensis Schu S4 has been shown to replicate within J774A.1 macrophage-like cells solely off host amino acids through autophagy-dependent nutrient acquisition (14). Transition metals, such as iron, have also been shown to be necessary for Francisella replication. Schu S4 acquires iron from the host cell through both a siderophore for ferric iron uptake and a ferrous iron transport system (15). A double mutant defective in both Francisella acquisition systems is unable to grow within macrophages and is attenuated in virulence (16, 17), suggesting that acquisition of transitional metals plays a key role in intracellular growth. However, the contribution of other trace metals, such as zinc, has not been characterized during Francisella infection. A better understanding of specific nutrient requirements and mechanisms of nutrient acquisition has the potential to identify targets for antimicrobials or potential vaccine candidates.

The transition metal zinc is an essential nutrient for both prokaryotic and eukaryotic cells. In prokaryotes, 4 to 8% of proteins bind zinc, which acts predominantly as a structural component of enzymes (18). While zinc is necessary for survival, excess zinc levels can also be detrimental by inducing toxicity through competition with other essential metals (19). In addition to maintaining zinc homeostasis under normal conditions, pathogens must also combat sequestration of zinc by host cells, a process known as nutritional immunity (20). This is especially important for intracellular pathogens like Francisella, as host cells have a number of mechanisms for controlling free zinc levels intracellularly. Host cells express zinc transporters that can transfer zinc between different intracellular compartments, limiting its availability in the phagosome, as well as the cytosol (21). They also express metallothioneins, proteins that tightly bind and chelate zinc, making free zinc scarce in the intracellular environment (22). Zinc transporter and metallothionein expression is regulated by proinflammatory cytokines (23, 24), indicating that zinc availability in the intracellular environment is tightly controlled during infection. To overcome these host defenses, bacteria must be able to regulate their intracellular zinc homeostasis in response to the host environment.

Maintenance of bacterial intracellular zinc homeostasis is mediated primarily through the expression of zinc importer and exporter proteins. Two major zinc transport proteins are involved in zinc uptake in most Gram-negative bacteria. The first is the zinc/iron permease (ZIP) family zinc transporter ZupT, which is a low-affinity transporter with broader specificity for zinc and other metals (25). In most bacteria, *zupT* is constitutively expressed, allowing low-level zinc transport regardless of bacterial zinc levels. In Salmonella enterica serovar Typhimurium, deletion of *zupT* alone does not cause any growth defect under zinc limitation; however, disruption of both *zupT* and the high-affinity *znuABC* zinc transport system genes results in a more severe defect in growth under zinc limitation than *znuABC* deletion alone (26). The ZnuABC transporter is the best-characterized system of high-affinity zinc import. It is composed of a periplasmic zinc-binding protein (ZnuA), a transmembrane protein (ZnuB), and an ATPase (ZnuC). The expression of zinc transporters is critical for bacterial survival in zinc limitation, as well as during infection within the host (27–29).

Expression of high-affinity importers and exporters is often tightly controlled by transcription factors that directly bind to zinc (30). The best-characterized regulator of zinc importer expression is the zinc uptake regulator (Zur). Zur is a member of the ferric uptake regulator (Fur) family of transcription factors that, when bound directly to transitional metals, bind to a specific sequence upstream of genes required for metal uptake and repress their expression (31). When intracellular zinc levels are low, Zur no longer binds zinc, causing Zur to undergo a conformational change. This conformational change prevents Zur binding to DNA, leading to the expression of genes required for zinc uptake and homeostasis. Zur-regulated genes can play a critical role in resistance to host zinc sequestration, as a number of bacterial mutants defective in Zur-regulated import proteins are impaired in zinc uptake *in vitro* and attenuated in virulence (27, 32). Therefore, identification of Zur-regulated genes can give a keen insight into mechanisms of zinc acquisition in bacterial species, as well as provide targets for attenuation of pathogens.

This work identified candidate genes for zinc uptake and maintenance of zinc homeostasis, as well as the atypical aspects of Zur and zinc regulation in Francisella. The F. novicida Zur ortholog was verified as a zinc-responsive transcription factor. Unexpectedly, only three genes were identified as regulated by F. novicida Zur, and of these, only one transporter was identified, the zinc permease-encoding gene *zupT*, which is typically constitutively expressed in other bacteria. In contrast, a high-affinity *znuACB* operon was found to be present in F. novicida but the expression of this operon was not controlled by Zur or zinc concentration. An F. novicida *zupT*::TN mutant was delayed in growth under zinc limitation but eventually grew to wild-type levels. This work also provides evidence for differences in zinc acquisition between F. novicida U112 and the more virulent F. tularensis subsp. tularensis Schu S4. In Schu S4, *zupT* is a pseudogene and *znuA* appears to be essential. Using a reverse TetR repression system, *znuA* expression in Schu S4 was repressed, revealing that *znuA* is required for growth under zinc limitation and contributes to intracellular growth within macrophages.

## RESULTS

### Identification of a Zur ortholog in Francisella.

F. novicida FTN_0881 encodes a 143-amino-acid protein (accession number ABK89769.1) that is annotated as a Fe^2+^/Zn^2+^ uptake regulator. Comparisons to the Zur proteins of Escherichia coli (accession number AAC77016.2) and Salmonella Typhimurium (accession number AAL56650.1) showed that the FTN_0881 protein has 36% and 35% identity, respectively, as well as 52% similarity, to both proteins. The FTN_0881 protein is predicted to have an N-terminal winged helix domain for DNA binding, as well as a C-terminal dimerization domain, both of which are conserved in Fur family proteins (31). The FTN_0881 protein also has conserved residues for the coordination of zinc at each of the three zinc-binding sites, including conserved residues D64, C80, H88, and H90 at site 2, which distinguish Zur orthologs from other Fur family proteins (31). On the basis of these features, the designation *zur* is proposed for the gene that encodes this protein.

A *zur* transposon mutant strain from an F. novicida U112 transposon library (3) was used to identify Zur-regulated genes. In the absence of Zur, expression of Zur-regulated genes should be derepressed, resulting in increased expression of these genes in a *zur* mutant strain. Transcriptome sequencing (RNA-Seq) was performed with RNA from wild-type and *zur*::TN mutant bacteria to investigate gene expression. Only five protein-encoding genes were differentially regulated (adjusted *P* value of <0.05) in the *zur*::TN mutant compared to the wild type (Table 1). In other bacteria, Zur typically regulates the expression of around 15 to 40 genes (31), although some Zur orthologs have been shown to regulate over 100 (33).

**TABLE 1:** Proteins whose genes are differentially expressed in a *zur*::TN mutant strain versus wild-type U112

Locus	Protein annotation^*a*^	Fold change	Adjusted *P* value
FTN_0880	Hypothetical protein	13.09	0
FTN_1758	Hypothetical protein	8.0	2.88E−244
FTN_1759	Hypothetical protein	2.71	8.46E−74
FTN_0879	ZIP family protein	1.26	0.000177
FTN_0395	ArsR family transcriptional regulator	1.27	0.00116

a Annotations as designated by NCBI.

The five F. novicida genes identified by RNA-Seq are shown in Table 1, and their organization is illustrated in Fig. S1 in the supplemental material. The genes for FTN_0880 and FTN_0879 appeared to be located in an operon and are transcribed off the opposite strand as *zur*. Cotranscription of the genes for FTN_0880 and FTN_0879 was confirmed by reverse transcription (RT)-PCR (Fig. S2). The FTN_0880 protein has similarity to the COG0523 family of P-loop GTPases, which includes conserved Walker A and B site residues, as well as a conserved CXCC motif for metal binding. This family is not well characterized but has been implicated in both metallochaperone activity and insertase activity of zinc into zinc-binding proteins (34). Recently, a COG0523 homologue in Acinetobacter baumannii was shown to be required for full growth under zinc limitation and to contribute to the maintenance of the labile pool of intracellular zinc (35), highlighting the role of some COG0523 family proteins in the maintenance of zinc homeostasis. The FTN_0879 protein has a conserved ZIP family domain, commonly found in a family of bacterial zinc permeases referred to as ZupT (36). ZupT is reported to act as a low-affinity zinc transporter with a broader metal specificity and is typically constitutively expressed regardless of the intracellular zinc concentration (25). While the predicted FTN_0879 protein has low sequence similarity to ZupT proteins of E. coli and Salmonella Typhimurium (around 35% identity over a 50-amino-acid stretch of the ∼250-amino-acid protein), the designation of the gene for FTN_0879 as *zupT* is proposed on the basis of the data below.

Three additional genes were also identified by RNA-Seq. FTN_1758 encodes a hypothetical protein that contains a DUF1826 domain, which is found in predicted succinylglutamate desuccinylase enzymes of other bacteria. This family of proteins may be zinc dependent (37); another DUF1826-containing protein in Pseudomonas fluorescens is downstream of a predicted Zur-binding site (34), suggesting that the gene may be Zur regulated. The gene for FTN_1759 is a nearby gene in the opposite orientation from FTN_1758. There are no conserved domains in the predicted protein, and it is only similar to other orthologs in different Francisella species by pBLAST. FTN_0395 is predicted to encode an ArsR family transcriptional repressor. ArsR family members repress the expression of genes required for metal export when metal concentrations are low and upregulate exporter gene expression when metal concentrations are high as a means to prevent toxicity (38). FTN_0395 is directly upstream of a predicted heavy metal P-type exporter, suggesting that FTN_0395 encodes a regulator of metal exporter expression.

### Zur directly regulates three genes in F. novicida.

**FIG 1:**
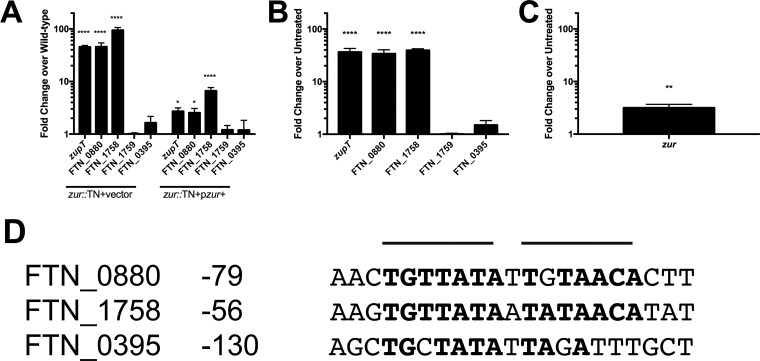
F. novicida Zur directly regulates three genes. (A, B) Expression of genes identified by RNA-Seq in either a *zur*::TN mutant carrying the empty vector (*zur*::TN+vector) and a complemented *zur*::TN mutant strain (*zur*::TN+p*zur*+) (A) or wild-type U112 in CDM supplemented with 10 μM TPEN (B). Results are shown as the fold difference in expression from that in the wild type grown in untreated CDM (three biological replicates). (C) Expression of *zur* in wild-type U112 grown in 10 μM TPEN-treated CDM. Results are shown as the fold changes in expression from that in the wild type grown in untreated CDM (three biological replicates). (D) Alignment of upstream regions of FTN_0880, FTN_1758, and FTN_0395. The values are distances from the predicted translational start site. Predicted heptameric palindromic repeats are overlined, while conserved nucleotides are in bold. Statistical analysis was performed by one-way ANOVA with Dunnett's multiple-comparison test (A, B) or Student's *t* test (C) compared to a reference value of 1.0. *, *P* < 0.05; **, *P* < 0.01; ****, *P* < 0.0001.

RNA-Seq results were validated by analyzing the expression of candidate genes in both the wild-type and *zur*::TN backgrounds by quantitative RT (qRT)-PCR. The expression of *zupT*, FTN_0880, and FTN_1758 genes was at least 30-fold greater in the *zur*::TN mutant than in the wild type (Fig. 1A). FTN_0395 was slightly (2-fold) upregulated in the *zur*::TN mutant, while FTN_1759 showed no difference in expression between the wild-type and *zur*::TN mutant strains. Complementation of the *zur* gene in *trans* partially restored the expression of upregulated genes to wild-type levels, indicating that the phenotype observed is due to the loss of *zur* and not to polar effects on downstream genes. Similar results were obtained when looking at gene expression in wild-type F. novicida grown in either complete Chamberlain's defined medium (CDM) alone or supplemented with 10 μM *N*,*N*,*N*′,*N*′-tetrakis(2-pyridylmethyl)ethylenediamine (TPEN), a zinc chelator. The expression of *zupT*, FTN_0880, and FTN_1758 genes was highly upregulated (>30-fold) under the zinc-limiting condition compared to that in untreated samples (Fig. 1B), while neither FTN_0395 nor FTN_1759 was differentially expressed under these conditions, indicating that these genes are not regulated by changes in zinc concentration. The expression of *zur* was also significantly increased during zinc limitation (Fig. 1C), which is consistent with the literature reporting that most *zur* genes are autoregulated (31). This result supports the identification of the gene for FTN_0881 as *zur*, a zinc-responsive transcription factor gene, and implicates three genes as potentially involved in the Zur-mediated response to zinc limitation.

Zur binds to a conserved inverted palindromic repeat sequence upstream of regulated genes to repress their transcription (31). It is likely that identified Zur-regulated genes also have a consensus Zur-binding site upstream of their translational start sites. Multiple-sequence alignment with Clustal Omega (39) identified a Zur consensus binding site in the upstream regions of genes with increased expression by qRT-PCR in the *zur*::TN mutant strain (FTN_0880, FTN_1758, and FTN_0395). As *zupT* and FTN_0880 are cotranscribed, only the upstream region of FTN_0880 was used for the multiple-sequence alignment. A palindromic sequence with similarity to other Zur-binding sequences (40) was identified upstream of the predicted FTN_0880 and FTN_1758 translational start sites (Fig. 1D). As *zur* shares an upstream promoter region with FTN_0880, this binding site likely also explains *zur* autoregulation. The promoter region of FTN_0395 had some sequence similarity to the first repeat, but this motif was found farther upstream (130 bp) from the initial ATG start site than in FTN_0880 (79 bp) and FTN_1758 (56 bp), which were more typical locations for Zur-binding sites. The upstream promoter region of FTN_1759 did not contain any sequences with similarity to a Zur-binding site. These data support direct Zur regulation of *zupT*, FTN_0880, and FTN_1758 genes, as well as further support the idea that Zur does not directly regulate FTN_0395 or FTN_1759.

### F. novicida contains *znuACB* homologues that are not regulated by Zur.

Only one of the genes identified as Zur regulated is annotated as having transporter activity. This gene, *zupT* (FTN_0879), encodes a ZIP family protein that acts as a low-affinity zinc transporter in other bacteria. No high-affinity transporter homologs were identified as Zur regulated by RNA-Seq, suggesting that these genes are either absent from the F. novicida genome or regulated by a Zur-independent mechanism. The F. novicida genome was bioinformatically analyzed for genes showing similarity to known zinc transporters, and the genes for FTN_0181 to FTN_0183, with limited similarity to the high-affinity zinc transport complex *znuACB*, were identified. PCR was performed with cDNA generated from F. novicida, and it was confirmed that these genes are located in an operon (Fig. S3). On the basis of the data below, the designation of this operon as *znuACB* is proposed.

**FIG 2:**
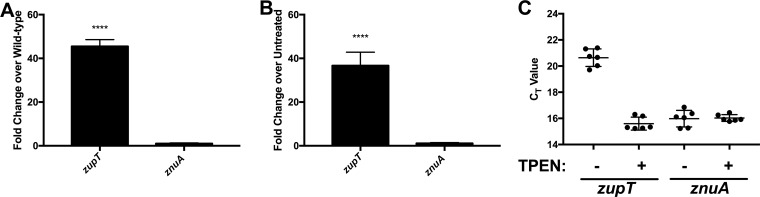
Expression of *znuA* is not regulated by Zur, but *znuA* is highly expressed. (A, B) Expression of *zupT* and *znuA* in either a *zur*::TN mutant strain grown in complete CDM (A) or wild-type U112 grown in CDM supplemented with 10 μM TPEN (B). Results are shown as the fold changes in expression from that in the wild type grown in untreated CDM (three biological replicates). (C) *C_T_* values for *zupT* or *znuA* from wild-type U112 grown in untreated or 10 μM TPEN-treated CDM. Statistical analysis was performed by one-way ANOVA with Dunnett's multiple-comparison test by using a reference value of 1.0. ****, *P* < 0.0001.

To better understand the regulation of this operon in response to zinc, *znuA* transcript expression was analyzed by qRT-PCR. As previously described, expression of the F. novicida *zupT* homolog is increased >30-fold in a *zur*::TN mutant or during zinc limitation (Fig. 1A and B). In contrast, no difference in *znuA* expression was identified by qRT-PCR under either of these conditions (Fig. 2A and B), suggesting that *znuA* expression does not respond to differences in zinc concentration. While no differences in expression were observed between untreated and TPEN-treated conditions, *znuA* transcript levels were estimated by comparing qRT-PCR *C_T_* values, the point at which fluorescence due to amplification surpasses a threshold level. Lower *C_T_* values correlate with higher gene transcript levels. *C_T_* values for *znuA* were similar under both conditions to *zupT C_T_* values in the presence of TPEN (Fig. 2C), suggesting that while *znuA* expression does not respond to changes in the zinc concentration, it is homeostatically expressed at high levels and may play a biologically important role in Francisella zinc uptake.

### F. novicida *zupT* facilitates growth under zinc limitation.

**FIG 3:**
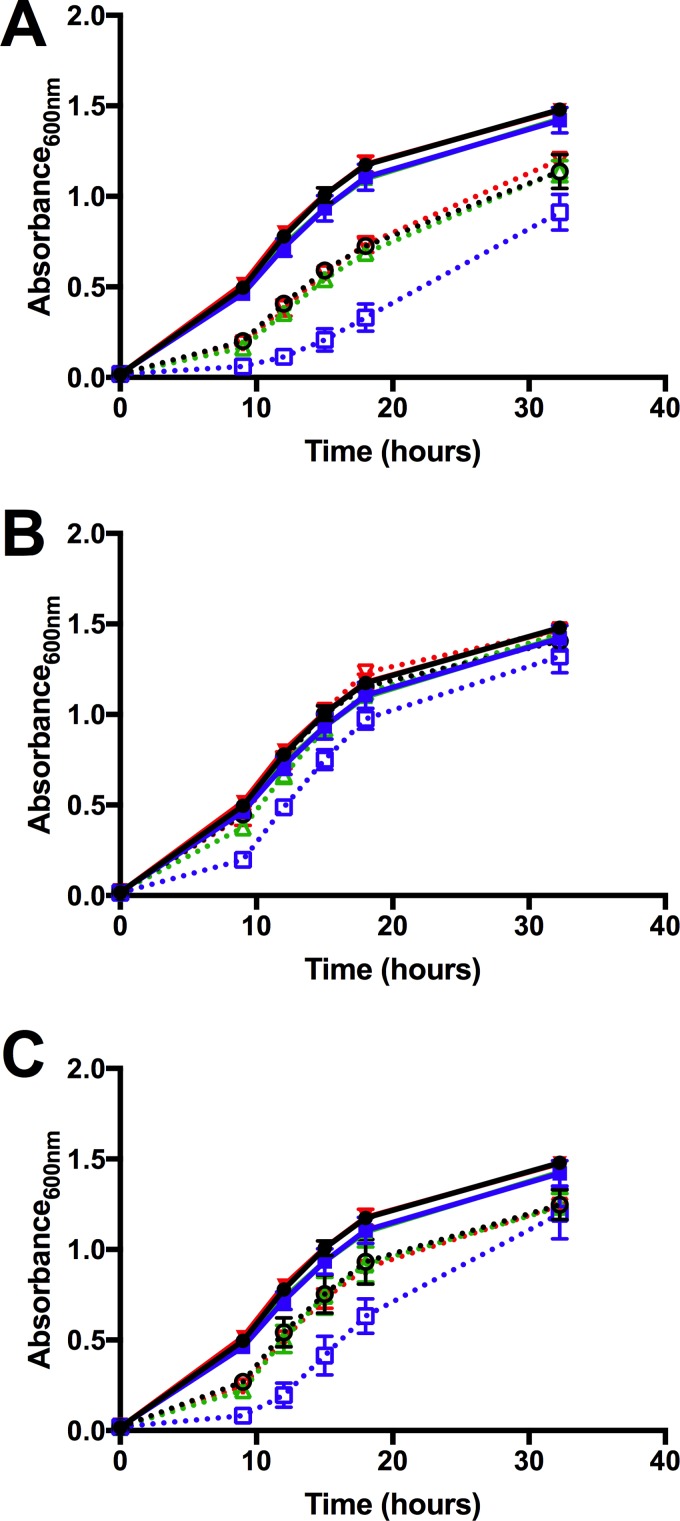
*zupT*::TN growth is significantly delayed during zinc limitation. The growth of the wild type plus vector (black), a *zupT*::TN mutant plus the vector (blue), a *znuA*::TN mutant plus vector (red), and a *zupT*::TN mutant plus p*zupT* (green) was measured by taking OD_600_ readings of TPEN-starved cultures over time. The growth of strains in complete medium (solid lines) is shown in all panels for comparison. Strains were grown (dotted lines) in CDM supplemented with 10 μM TPEN (A), CDM supplemented with 10 μM TPEN and 2 μM ZnSO_4_ (B), or CDM supplemented with 10 μM TPEN and 2 μM MnCl_2_ (C). Data are presented as the average of three separate experiments.

If *zupT* and *znuACB* contribute to zinc uptake in F. novicida, single transposon mutant strains should have impaired zinc uptake compared to that of the wild type, which should affect their ability to grow under zinc limitation. To investigate the contribution of these transporters, the growth of single *zupT* and *znuA* transposon mutants was examined under zinc-limiting conditions. No difference in growth between wild-type and single transposon mutant strains grown in untreated and TPEN-treated CDM was observed (data not shown), possibly due to the utilization of intracellular zinc stores. To test this, the wild-type and mutant strains were first starved overnight in TPEN-treated CDM to exhaust their intracellular zinc stores and then diluted in either complete or TPEN-treated CDM to test bacterial growth. The growth of a *zupT*::TN mutant strain was significantly delayed compared to that of the wild type in the presence of TPEN (Fig. 3A). Complementation of this strain in *trans* resulted in the restoration of growth to wild-type levels. The growth of the *zupT*::TN mutant was also restored by the addition of 2 μM ZnSO_4_ to TPEN-treated cultures (Fig. 3B). Supplementation with 2 μM MnCl_2_ failed to restore growth (Fig. 3C), suggesting that the growth defect observed was specifically due to zinc limitation. No difference in growth between the *znuA*::TN mutant strain and the wild type in TPEN-treated medium was observed (Fig. 3A), suggesting that ZnuA may not play a major role in zinc transport in F. novicida or may transport metals other than zinc.

### *znuA* is essential in F. tularensis subsp. tularensis Schu S4.

**FIG 4:**
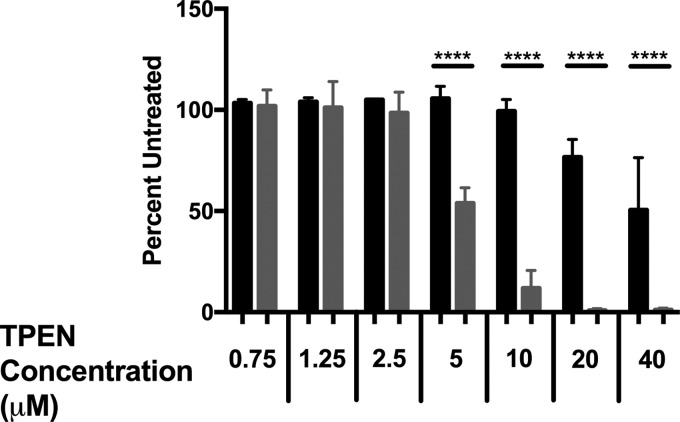
F. tularensis Schu S4 is more sensitive to zinc limitation than F. novicida U112. The growth of either wild-type U112 (black) or Schu S4 (gray) in increasing concentrations of TPEN after 24 h is shown. Growth is presented as the percentage of untreated U112 or Schu S4, respectively. Data are presented as the average of three separate experiments. Statistical analysis was performed by one-way ANOVA with Tukey's multiple-comparison test. ****, *P* < 0.0001.

Although F. novicida is used as a model for more virulent F. tularensis subsp. tularensis strains, it has several differences that make comparisons between species difficult. Most notably, the more virulent strains are thought to have gained virulence in part by gene loss and are enriched in pseudogenes, many in metabolic pathways (6, 41). This gene loss is observed in zinc-regulated genes as well. Of the three identified Zur-regulated genes in F. novicida, only one (FTN_0880) is thought to encode a functional protein in the F. tularensis subsp. tularensis Schu S4 strain (FTT1000). Notably, the *zupT* gene is disrupted by a premature stop codon in Schu S4, indicating that it is likely nonfunctional. While a *znuA*::TN mutant of U112 did not exhibit a growth defect under zinc limitation, the loss of *zupT* in Schu S4 suggests a more prominent role for the ZnuABC transporter in this strain. Loss of *zupT* and other Zur-regulated genes likely impairs the ability of Schu S4 to acquire zinc and establish intracellular zinc stores, as Schu S4 exhibits greater sensitivity to zinc limitation than the F. novicida U112 strain (Fig. 4) and is incapable of growth under zinc limitation after overnight zinc starvation (data not shown).

As *znuACB* is likely the primary mechanism of zinc uptake in the virulent Schu S4 strain, the construction of a clean *znuA* deletion strain was attempted using an allelic replacement technique that has been previously described (42, 43). Briefly, a suicide plasmid containing the upstream and downstream regions of *znuA* was introduced into Schu S4, leading to homologous integration into the genome. A recombination event and subsequent loss of the suicide plasmid should result in strains that randomly maintained either the genomic *znuA* copy or a clean deletion of *znuA*. Despite multiple attempts, a clean deletion strain was not obtained by this method. Further investigation determined that although the plasmid containing upstream and downstream regions of *znuA* was incorporated into the genome, recombination always favored maintaining the genomic *znuA* copy over the clean deletion of *znuA*, suggesting that there is strong selective pressure to maintain the *znuA* gene in Schu S4. To investigate the role of *znuA* in Schu S4, an inducible repression system (a kind gift from Tom Kawula) was used (44). The *znuA* gene was introduced into a Francisella plasmid so that *znuA* would be under the control of a promoter regulated by reverse TetR (RevTet) repression. Expression is turned off after the addition anhydrotetracycline (ATc), a tetracycline analogue that lacks antibiotic activity. A wild-type Schu S4 strain carrying the RevTet *znuA* plasmid was created, and then a genomic *znuA* deletion strain was generated in the presence of the RevTet *znuA* construct by the introduction of the *znuA* suicide plasmid as described above, resulting in a strain in which *znuA* expression can be repressed (Δ*znuA* p*znuA* mutant).

**FIG 5:**
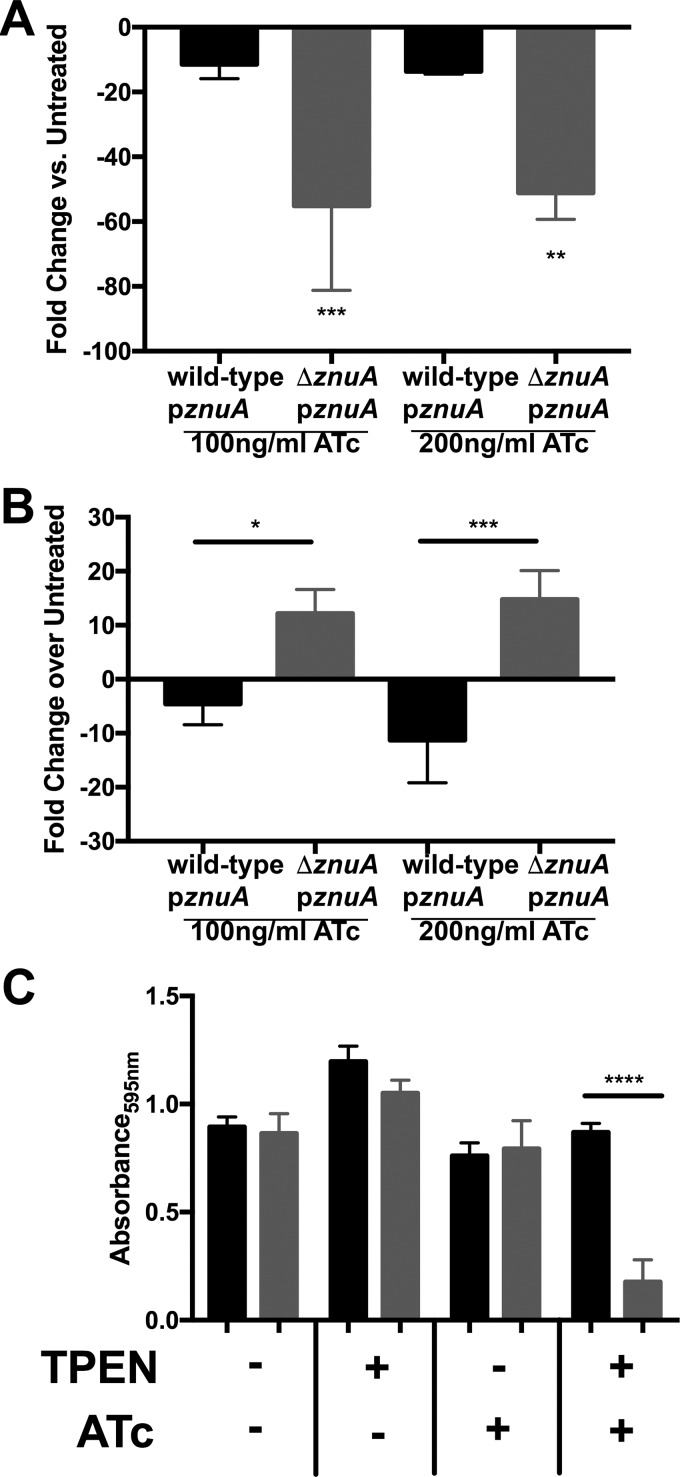
Growth of the Δ*znuA* p*znuA* mutant is impaired in the presence of both ATc and TPEN. (A) Expression of *znuA* in either the wild type containing p*znuA* or the Δ*znuA* p*znuA* mutant grown in Che-CDM supplemented with ATc at either 100 or 200 ng/ml. Results are shown as the fold change in expression over the wild-type strain containing p*znuA* or the Δ*znuA* p*znuA* mutant strain grown in untreated Che-CDM (three biological replicates). (B) Expression of FTT1000 in either the wild type containing p*znuA* or the Δ*znuA* p*znuA* mutant grown in Che-CDM supplemented with ATc at either 100 or 200 ng/ml. Results are shown as the fold changes in expression from that in the wild type containing p*znuA* or the Δ*znuA* p*znuA* mutant strain grown in untreated Che-CDM (three biological replicates). (C) Growth of either the wild type containing p*znuA* (black) or the Δ*znuA* p*znuA* strain (gray) in either untreated Che-CDM or Che-CDM supplemented with 1.25 μM TPEN, 100 ng/ml ATc, or both TPEN and ATc after 24 h. Strains in ATc were grown for 24 h in Che-CDM supplemented with 100 ng/ml ATc prior to the growth assay. Data are presented as the average of three separate experiments. Statistical analysis was performed by one-way ANOVA with Dunnett's multiple-comparison test. *, *P* < 0.05; **, *P* < 0.01; ***, *P* < 0.001; ****, *P* < 0.0001.

Growth assays performed with the Δ*znuA* p*znuA* mutant in complete CDM produced variable results, possibly due to small variations in zinc within the medium. To control for medium zinc concentrations, CDM was first Chelex treated to chelate metals before adding metals back at defined concentrations. Chelex-treated CDM (Che-CDM) was then stored in acid-washed glassware to prevent leeching of zinc into the medium. Wild-type Schu S4 containing p*znuA* and the Δ*znuA* p*znuA* mutant were first grown in Che-CDM supplemented with ATc to allow for ZnuA protein turnover. After 24 h of growth in ATc, bacteria were diluted in Che-CDM with ATc and qRT-PCR was used to confirm *znuA* repression relative to its expression in untreated Che-CDM. The expression of *znuA* was significantly lower in the Δ*znuA* p*znuA* mutant with the addition of ATc than under untreated conditions (Fig. 5A), indicating that ATc treatment successfully represses plasmid *znuA* expression. A Schu S4 strain expressing a RevTet construct encoding a hemagglutinin-tagged version of *znuA* was generated, and analysis of *znuA* protein levels by Western blotting also showed decreased protein expression after ATc treatment (Fig. S4). However, deletion of the genomic *znuA* gene from this strain was unsuccessful, possibly due to inefficient function of the tagged protein.

If the ZnuABC transporter is a primary mechanism of zinc acquisition in Schu S4, the Δ*znuA* p*znuA* mutant strain should also be impaired in the ability to acquire zinc. To estimate intracellular zinc levels, expression of the Zur-regulated FTT1000 gene (FTN_0880) in the presence of ATc was compared with that in its absence. If the intracellular zinc concentration is low, Zur-regulated genes should be derepressed and FTT1000 gene expression should increase. The expression of FTT1000 was at least 10-fold greater in the Δ*znuA* p*znuA* mutant treated with ATc than in the wild type with p*znuA* treated with ATc (Fig. 5B), suggesting that repression of *znuA* expression results in decreased intracellular zinc.

### *znuA* is required for Schu S4 growth under zinc limitation.

The effect of *znuA* expression on Schu S4 growth was characterized by comparing the growth of the wild type containing p*znuA* and the Δ*znuA* p*znuA* mutant strain in the presence and absence of ATc as described above. No difference in growth was observed between the wild type containing p*znuA* and the Δ*znuA* p*znuA* mutant strain in either untreated Che-CDM or Che-CDM supplemented with ATc (Fig. 5C). If the product of *znuA* is acting as a zinc transporter, the Δ*znuA* p*znuA* mutant should be unable to grow under zinc limitation when *znuA* expression is turned off. To test this, the growth of the Δ*znuA* p*znuA* mutant in the presence of 1.25 μM TPEN was compared with that in its absence. In bacteria that were not treated with ATc, no difference between the growth of the wild type containing p*znuA* and that of the Δ*znuA* p*znuA* mutant strain was observed. However, when bacteria were first pretreated with ATc and then grown in Che-CDM supplemented with both ATc and TPEN, the wild type containing p*znuA* grew normally while the Δ*znuA* p*znuA* mutant failed to grow (Fig. 5C). These data suggest that while treatment of the Δ*znuA* p*znuA* mutant with ATc to repress *znuA* expression or with TPEN to limit the zinc concentration does not affect bacterial growth, combination treatment greatly impairs bacterial growth. The decreased intracellular zinc, as measured by the increase in FTT1000 expression, and the inability of the Δ*znuA* p*znuA* mutant strain to grow in the presence of ATc and zinc limitation support the idea that ZnuABC is a primary mechanism of zinc uptake in Schu S4.

### Growth of the Δ*znuA* p*znuA* mutant in J774A.1 macrophage-like cells is impaired.

The role of Francisella zinc uptake during intracellular growth has not been characterized. As Francisella quickly escapes from the phagosomal compartment to reside and grow within the host cell cytosol, limitation of zinc in this environment would likely have the greatest impact on Francisella growth.

**FIG 6:**
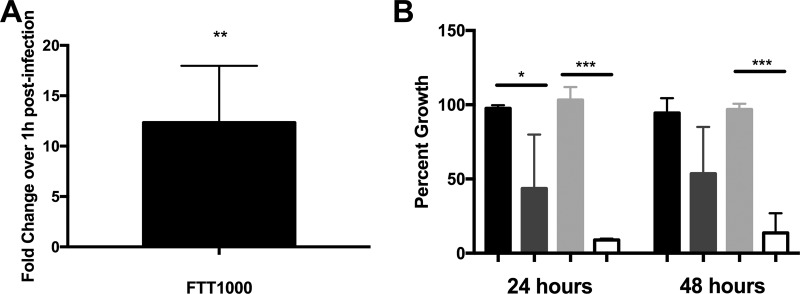
The Δ*znuA* p*znuA* mutant is attenuated in intracellular growth in J774A.1 cells. (A) Expression of FTT1000 in wild-type Schu S4 grown in J774A.1 cells at 8 h postinfection. Results are shown as the fold changes in expression from that in the wild type at 1 h postinfection. (B) Percent growth of the untreated wild type containing p*znuA* (black bars), the untreated Δ*znuA* p*znuA* mutant (dark gray bars), the wild type containing p*znuA* pretreated with 100 ng/ml ATc (light gray bars), or the Δ*znuA* p*znuA* mutant strain pretreated with 100 ng/ml ATc (white bars) in J774A.1 cells. Data are the percent growth of each strain compared to that of the wild type either untreated or treated with ATc at 100 ng/ml (three biological replicates). Statistical analysis was performed with a Student *t* test against a reference value of 1.0 (A) or one-way ANOVA with Tukey's multiple-comparison test (B). *, *P* < 0.05; **, *P* < 0.01; ***, *P* < 0.0001.

To better understand the effect of the host cell cytosol on Francisella intracellular zinc levels, expression of the Zur-regulated FTT1000 gene was observed as a marker of zinc limitation during wild-type Schu S4 growth in J774A.1 macrophage-like cells. qRT-PCR showed that FTT1000 was significantly upregulated at 8 h postinfection (Fig. 6A), a time point when bacterial replication has likely begun, suggesting that the host cell cytosol is a zinc-limiting environment. Similar upregulation of FTT1000 was observed in primary bone marrow-derived macrophages (data not shown). To characterize the significance of ZnuABC for intracellular replication, J774A.1 macrophage-like cells were infected with either p*znuA*-containing wild-type bacteria or Δ*znuA* p*znuA* mutant bacteria that were either untreated or pretreated with ATc at 100 ng/μl. The growth of ATc-treated Δ*znuA* p*znuA* mutant bacteria was significantly less than that of the ATc-treated the wild type containing p*znuA* at both 24 and 48 h postinfection (Fig. 6B). The growth of untreated Δ*znuA* p*znuA* mutant bacteria varied across experiments but trended lower than that of the untreated wild type containing p*znuA*. These data suggest that *znuA* plays an important role in overcoming the zinc-limiting environment of the host cell cytosol to facilitate bacterial growth.

## DISCUSSION

Acquisition of zinc is critical for survival in the intracellular environment. Better understanding of regulatory mechanisms can potentially identify genes that are necessary for pathogen survival. In this study, RNA-Seq was performed to identify Zur-regulated genes in F. novicida U112. Five genes were identified by RNA-Seq as significantly upregulated in a *zur*::TN mutant compared to the wild type, and three of them were confirmed by qRT-PCR as upregulated both in a *zur*::TN mutant and during zinc limitation. While the FTN_0395 gene was slightly upregulated in a *zur*::TN mutant, it did not show differences in expression in response to the zinc concentration and did not contain a consensus Zur-binding site, suggesting that it is not directly regulated by Zur. The slight increase in FTN_0395 gene expression in the *zur*::TN strain could be due to disruption of zinc homeostasis in the absence of Zur. The expression of an ArsR family transcriptional regulator was similarly increased in an A. baumannii *zur*::TN mutant (33). The FTN_1759 gene was also identified by RNA-Seq but showed no difference in expression either in a *zur*::TN mutant or under zinc limitation. Detection of this gene may be due to the expression of FTN_1758, which is highly upregulated and in close proximity to the gene.

The identification of only three Zur-regulated genes was unexpected, as analyses of Zur-regulated genes in other bacteria have identified regulons ranging from 15 to 100 genes (31, 33). Genes upregulated in the F. novicida *zur*::TN mutant were also upregulated under zinc limitation, and the upstream region of these genes contained predicted Zur-binding sequences (Fig. 1), indicating that while regulation by Zur in Francisella functions similarly to that in other bacteria, the Francisella Zur regulon is reduced. One possible explanation is that the Francisella genome has pared the Zur regulon down to only those genes that are essential for growth under zinc limitation. However, the fact that *znuACB*, which is important for growth under zinc limitation, is present in Francisella but unregulated by Zur (Fig. 2) indicates that Francisella Zur-regulated genes are not the only genes required for growth under zinc limitation. Rather, these data indicate that regulation of zinc uptake by Zur or other regulatory mechanisms is unnecessary, possibly due to Francisella adaptation to zinc-poor environments.

The Francisella *zupT* gene was the only identified Zur-regulated gene annotated as encoding a metal transporter. Regulation of *zupT* by Zur was unexpected, as characterized *zupT* homologs are not Zur regulated and are typically constitutively expressed. The only observed Zur-regulated *zupT* homolog is from *Cupriavidus metallidurans* (45), a bacterium that is adapted to growth in a high-metal environment and lacks a ZnuABC-type zinc transporter, making ZupT the primary zinc uptake mechanism. Repression of Francisella *zupT* expression under zinc-replete conditions may indicate differences in the affinity of Francisella ZupT for zinc and suggest that *zupT* plays a larger role in zinc acquisition in F. novicida than in other bacteria. This is also supported by the delayed growth of an F. novicida *zupT*::TN mutant observed under zinc-limiting conditions, as *zupT* mutant strains of other bacteria often have no phenotype unless they also have mutations in other zinc transporters (26, 46). The Francisella ZupT protein has around 35% identity over a 50-amino-acid stretch of the ∼250-amino-acid protein, supporting the possibility that there are structural differences that enhance zinc affinity. It was also unusual that *znuACB* expression was not regulated by Zur (Fig. 2). The *znuACB* operon is often regulated by Zur in other bacteria (32, 47, 48), presumably to regulate its expression and prevent zinc toxicity in a zinc-replete environment. The Francisella Zur regulon is reduced compared to that in other bacteria, so this may just be a reflection of the minimized role of Zur in zinc-dependent regulation or of regulation of zinc-dependent genes in general. It may also reflect a restriction of Francisella to zinc-poor environments, making it unnecessary to regulate the expression of a high-affinity zinc importer.

These data suggest that F. novicida U112 has a greater capacity than F. tularensis subsp. tularensis Schu S4 to adapt to zinc limitation, which may reflect the genomic decay that has occurred in the pathogenic F. tularensis subspecies. Wild-type Schu S4 is more sensitive to zinc limitation than U112 (Fig. 4), which may be due to redundant mechanisms of acquiring zinc or adapting to zinc limitation. Multiple Zur-regulated genes are absent from or nonfunctional in Schu S4 compared to U112, potentially leading to compensation of these roles by remaining proteins. A *znuA*::TN mutant of U112 grows similarly to the wild type under zinc limitation, and while the growth of a *zupT*::TN mutant was delayed, it eventually reached wild-type levels (Fig. 3). In Schu S4, where *zupT* is a pseudogene, a Δ*znuA* p*znuA* mutant failed to grow after 24 h under zinc limitation (Fig. 5), indicating that ZnuABC may take on the role of both transporters in this strain. F. tularensis and F. novicida occupy distinct ecological niches, as F. tularensis is a zoonotic pathogen propagated by an arthropod vector, while there is no evidence of zoonotic F. novicida infection in nature (6). Specialization by F. tularensis for the host environment has led to the loss of a number of metabolic pathways, such as amino acid biosynthesis and purine metabolism (1, 6, 41), which are no longer necessary for growth. Similarly, *zupT* and other Zur-regulated genes that are missing from Schu S4 are likely unnecessary for growth in the intracellular environment.

While many Zur-regulated genes were lost by Schu S4, *znuACB* was specifically maintained, indicating that it plays a more prominent role in zinc acquisition by the more virulent F. tularensis subspecies. This work suggests that there is strong selective pressure to maintain this operon, as attempts to delete *znuA* were unsuccessful in the absence of an in *trans znuA* copy. The growth of the Δ*znuA* p*znuA* mutant strain is more severely affected by zinc limitation than that of the U112 *znuA*::TN or *zupT*::TN mutant, indicating that the ZnuABC transporter may compensate for the loss of ZupT function in Schu S4. Of note, only one Zur-regulated gene, FTT1000, was maintained in Schu S4. Attempts to delete FTT1000 from Schu S4 were unsuccessful, similar to those with *znuA*, suggesting that there may also be pressure to maintain FTT1000 in Schu S4. The FTT1000 protein is a member of the COG0523 family of P-loop GTPases, which are hypothesized to have zinc chaperone and insertase activities. Mutation of a COG0523 family protein leads to attenuation of Brucella suis intracellular growth within macrophages (49) and dissemination in A. baumannii (35), suggesting that this family of proteins may contribute to virulence in other bacteria. As this family of proteins is not well characterized, further investigation of the function and role of FTT1000 in virulence is necessary.

Overall, this work characterizes the Zur regulon in F. novicida U112 and identifies multiple transporters that contribute to growth under zinc limitation. Examination of the Zur regulon highlights a reduced role in the regulation of zinc uptake in Francisella, potentially due to environmental adaptation to zinc-poor environments. This is observed further in the pathogenic F. tularensis subsp. tularensis Schu S4 strain, where only one Zur-regulated gene is maintained and the strain is more sensitive to zinc limitation (Fig. 4). The limited number of genes identified in response to zinc limitation suggests that the remaining mechanisms are highly efficient at zinc acquisition and homeostasis and strongly maintained by the virulent strains. This work also highlights the need to validate Francisella research in more-virulent strains, as less-virulent strains such as those of F. novicida may possess redundant mechanisms or nutritional needs different from those of more-virulent strains.

## MATERIALS AND METHODS

### Bacterial strains and media.

F. novicida U112 and F. tularensis subsp. tularensis Schu S4 were grown on cysteine-supplemented Mueller-Hinton agar (MHA/c) plates. All experiments with the Schu S4 strain were performed in an approved biosafety level 3 laboratory. U112 single transposon mutants were obtained from the F. novicida transposon library (3). For liquid growth, the U112 and Schu S4 strains were grown in complete CDM or in Che-CDM where specified. Che-CDM was prepared by treating complete CDM overnight with 10 g/liter Chelex 100 at 4°C. Che-CDM was then filter sterilized and supplemented with FeSO_4_, MgSO_4_, and CaCl_2_. Acid-washed glassware was prepared by soaking glassware overnight in 10% metal grade HNO_3_ and then rinsing it five times with Milli-Q water. Plates and broth were supplemented with 200 μg/ml hygromycin or 15 μg/ml kanamycin when necessary for selection.

### Generation of mutant and complemented strains.

U112 complemented strains were generated with the pEDL56 vector, a kind gift from Tom Kawula (Washington State University). Briefly, DNA was amplified by PCR with the Phusion PCR polymerase (Thermo Fisher) and gene-specific primers. PCR products and the pEDL56 vector were digested with the restriction enzymes MluI-HF and XmaI (New England BioLabs) and then ligated with T4 DNA ligase (New England BioLabs). Ligated plasmids were introduced into subcloning efficiency DH5α cells (ThermoFisher) by chemical transformation and grown on LB plates supplemented with 200 μg/ml hygromycin to select for transformants. Plasmids were purified with the QIAprep spin miniprep kit (Qiagen). Isolated plasmids were introduced by electroporation into electrocompetent F. novicida MFN245, a kind gift from Colin Manoil and Larry Gallagher (University of Washington), with a Gene Pulser II (Bio-Rad) at 1.5 kV, 400 Ω, and 25 μF. Transformants were selected by growth on MHA/c plates supplemented with 200 μg/ml hygromycin. Plasmids were then isolated from MFN245 as described previously and introduced into electrocompetent wild-type F. novicida U112 or selected transposon mutants.

### Construction of a *znuA* deletion strain.

Before deletion of the Schu S4 *znuA* gene (FTT0209), a pEDL56 vector containing the Schu S4 *znuA* gene was constructed as described above. The construct was introduced into electrocompetent wild-type Schu S4 with a Gene Pulser II at 2 kV, 400 Ω, and 25 μF. Transformants were selected by growth on MHA/c plates supplemented with 200 μg/ml hygromycin. To construct an in-frame deletion of *znuA*, DNA sequences upstream and downstream of *znuA* were amplified by PCR and introduced into the *sacB* suicide vector pGIR463, a kind gift from Girija Ramakrishnan. This plasmid was introduced by electroporation into Schu S4/pEDL56::*znuA*, and integrant and single-deletion strains were selected as previously described (12).

### RNA isolation from bacterial cultures.

For RNA isolation, biological replicates of the strains indicated were grown overnight in complete CDM at 37°C. Overnight cultures were diluted to an optical density at 600 nm (OD_600_) or OD_595_ of 0.2 for RNA-Seq experiments or 0.01 for quantitative RT-PCR in 1 ml of CDM and grown at 37°C until mid-log phase. Bacteria were pelleted by centrifugation, and RNA was purified with the RNeasy kit (Qiagen). Genomic DNA contamination was removed with the Turbo DNase kit (Ambion). The total RNA concentration was then quantified with the NanoDrop 1000 spectrophotometer (Thermo Scientific).

### RNA-Seq.

RNA was isolated from three separate biological replicates of either wild-type or *zur*::TN mutant U112. RNA quality was confirmed with the Agilent Bioanalyzer at the Biomolecular Research Facility at the University of Virginia (UVA). Total RNA was submitted to HudsonAlpha (Huntsville, AL), where ribosomal reduction was performed to concentrate for mRNA. Directional cDNA libraries were then generated, and samples were sequenced in multiplex (50 paired-end reads) with the Illumina HiSeq v4 platform.

Data analysis was performed by the UVA Bioinformatics Core. Briefly, gene reads were first aligned with reference genomes and read counts were quantified with EDGE-pro software. The Deseq2 Bioconductor package was then used to normalize the read counts and estimate dispersion for each gene. These data were then fitted to a negative binomial model, which was used for differential-expression analysis. Genes were considered differentially expressed between groups when the adjusted *P* value was <0.05.

### cDNA generation and quantitative PCR.

Purified RNA samples were converted to first-strand cDNA with Moloney murine leukemia virus (M-MuLV) reverse transcriptase in accordance with the manufacturer's protocol. Briefly, a 20-μl reaction mixture was set up with 500 ng of purified RNA, 0.5 mM (each) deoxynucleoside triphosphate (New England BioLabs), 0.15 μg/μl random primers (Invitrogen), 10 mM dithiothreitol, 1× first-strand buffer (Invitrogen), and 200 U of M-MuLV reverse transcriptase (Invitrogen) in nuclease-free water. RT was performed by incubating the reaction mixture at room temperature for 10 min, at 37°C for 50 min, and then at 70°C for 15 min. Samples were diluted to a 100-μl total volume with nuclease-free water.

Quantitative PCR was performed with the SensiFAST SYBR and fluorescein kit (Bioline). Control reaction mixtures with no reverse transcriptase were used to ensure that each sample was free of genomic DNA contamination. Duplicate wells of three biological replicate samples were assayed for each gene and condition. Differential expression of each gene was calculated relative to the housekeeping gene *fopA* by the comparative *C_T_* method (50).

### Bacterial growth.

Bacterial strains from −80°C stocks were cultured on MHA/c plates. For bacterial growth assays, single colonies were inoculated into complete CDM or Che-CDM when specified and grown overnight at 37°C with shaking. Overnight cultures were diluted to an OD of 0.01 in CDM supplemented with 10 μM TPEN (Sigma) and grown overnight at 37°C with shaking. Overnight starved cultures were then diluted to an OD of 0.01 in CDM that was untreated or supplemented with 10 μM TPEN or with 10 μM TPEN and 2 μM ZnSO_4_ or MnCl_2_ (Sigma). The absorbance of each bacterial culture was determined by measuring the OD_600_ with a Genesys 20 visible spectrophotometer (Thermo Scientific) or the OD_595_ with an iMark microplate absorbance reader (Bio-Rad).

### Infection of J774A.1 macrophage-like cells with F. tularensis subsp. tularensis.

Overnight cultures of Schu S4/pEDL56::*znuA* or the Δ*znuA*/pEDL56::*znuA* mutant were grown in Che-CDM. Bacteria were diluted to an OD_595_ of 0.01 in Che-CDM that was either untreated or supplemented with 100 ng/ml ATc and grown for 24 h. Bacteria were diluted in Dulbecco's modified Eagle's medium (DMEM) and added to J774A.1 macrophage-like cells seeded into the wells of a 24-well plate at a multiplicity of infection of 50. The plate was centrifuged at 800 × *g* at 4°C for 10 min to bring the bacteria into contact with the J774A.1 cells and synchronize infections. The plate was then incubated at 37°C in 5% CO_2_ for 50 min to allow for phagocytosis. Cells were washed three times with phosphate-buffered saline (PBS) and then incubated with DMEM supplemented with 50 μg/ml gentamicin for 1 h to kill extracellular bacteria. After 1 h, cells were washed with PBS and incubated in complete DMEM at 37°C in 5% CO_2_. At the time points indicated, J774A.1 cells were lysed by treatment with 0.1% saponin for 5 min. Serial dilutions of the lysate were grown on MHA/c plates and incubated at 37°C, and bacterial colonies were counted after 2 days of incubation to enumerate the bacteria.

For RNA isolation, total RNA was recovered from infected cells at 1 and 8 h postinfection by aspirating off medium and adding 250 μl of TRIzol reagent to each well of the plate. Four replicate wells were pooled per sample. RNA was extracted by chloroform phase separation, and the aqueous layer was further purified with the RNeasy minikit (Qiagen) and the Turbo DNase kit (Ambion) as previously described.

### Statistical analysis.

Statistical analysis of RNA-Seq results was done as described above. For other experiments, data were analyzed with either a one-way analysis of variance (ANOVA) or a two-tailed Student *t* test by using Prism software (GraphPad Software, Inc., San Diego, CA).

## Supplementary Material

Supplemental material
