# Supplementary material for: Zinc Acquisition Mechanisms Differ between Environmental and Virulent Francisella Species
Source: J Bacteriol. 2018 Jan 24;200(4):e00587-17. doi: 10.1128/JB.00587-17 (PMC5786701; doi:10.1128/JB.00587-17)
Supplement: Supplemental material [file supp_200_4_e00587-17__index.html]

Supplemental material 

# Zinc Acquisition Mechanisms Differ between Environmental and Virulent Francisella Species

## Supplemental material

- Supplemental file 1 -

  Tables S1 (Oligonucleotides) and S2 (Strains and plasmids) and Fig. S1 (Genomic organization of candidate genes), S2 (Transcription of *zupT* and FTN\_0880 as an operon), S3 (Transcription of FTN\_0183-FTN\_0181 as an operon), and S4 (ATc repression of ZnuA-HA)

  PDF, 5.6M
