# Supplementary material for: Zinc Acquisition Mechanisms Differ between Environmental and Virulent Francisella Species
Source: J Bacteriol. 2018 Jan 24;200(4):e00587-17. doi: 10.1128/JB.00587-17 (PMC5786701; doi:10.1128/JB.00587-17)
Supplement: Supplemental material [file JB.00587-17_zjb004184631s1.pdf]

Supplemental Table 1: Oligonucleotides used in this study

Name	Sequence[a]	Source
zur complement 1-Fwd	TACAAAGGAGTAACTGCAGTTTGTTAATGAA TAAAAAT	This study
zur complement 1-Rev	GTCATCGTCTTTGTAGTCGATTTCTAAACATT T	This study
zur complement 2-Fwd	ATTCACGCGTACAAAGGAGTAACTGCAGTT	This study
zur complement 2-Rev	AATTCCCGGGTTACTTGTCGTCATCGTCTTT GTA	This study
zupT complement Fwd	ATTCACGCGTACAAAGGAGTAACTGCAGTAT GAGTAATTTTCAATTA	This study
zupT complement Rev	ATTACCCGGGCTACTTGTCGTCATCGTCTTT GTAGTCAGTCCAAATAGCAACTAT	This study
znuA pEDL56 Fwd	ATTCACGCGTACAAAGGAGTAACTGCAGTAT GAAAAAATACATACTA	This study
znuA pEDL56 Rev	ACTGCCCGGGTTATTTTTGTACTTTATCCAA	This study
znuA suicide vector 1 Fwd	ACTATCCGGATTTGGTGGTGGATTTGCTATT CTATGTGC	This study
znuA suicide vector 1 Rev	AAGGAAGACATATGCCAAAGCTACAATAACA ACAGCAGCTA	This study
znuA suicide vector 2 Fwd	ATCAACTGGCATATGGAAACATTACAAGCAA CTGCTGTGG	This study
znuA suicide vector 2 Rev	GTTAGGCTCGAGGCAATAAGATTTATAGCAA GTTGCCC	This study
fopA qPCR Fwd	CATCTATCGCTGCAGGTTCA	This study
fopA qPCR Rev	TCCAGCTAGACCGTTAGCATC	This study
zur qPCR Fwd	GCCGCTAAAGAATTTTGTGAA	This study
zur qPCR Rev	AGCCAAAAGTCTATCGCTCTG	This study
zupT qPCR Fwd	GGTGAAGCTTTAGCGAGTGG	This study
zupT qPCR Rev	TTGCCTTTATTGCTCCTTGA	This study
FTN_0880 qPCR Fwd	AATGCTCGACGGCAGAAAAA	This study
FTN_0880 qPCR Rev	GTTTCCGGCACATGCTCTTT	This study
FTN_1758 qPCR Fwd	GCAATGTGTCCTCGTTTCCA	This study

FTN_1758 qPCR Rev	CCATAACCAGAGTTGCCTATCCA	This study
FTN_1759 qPCR Fwd	TGCACCTTCTAATGCTCCAAAA	This study
FTN_1759 qPCR Rev	GCTGCAAATGGGGCTACAAA	This study
FTN_0395 qPCR Fwd	TCCAAAAAGCTTTGGGAGATGA	This study
FTN_0395 qPCR Rev	GCCAGTTTAGCTGCTGCTGGT	This study
znuA qPCR Fwd	CCCAAACAACAAAAGGCAGT	This study
znuA qPCR Rev	CATCTGCTTCGGCTAAAAGC	This study

[a]Primer sequences are provide as 5' to 3'

Supplemental Table 2: Strains and plasmids used in this study

	Genotype	Comments	Source
F. novicida U112	Wild-type	*F. novicida*	BEI Resources
NR-3065	*F. novicida zur::*TN		(1)
NR-8039	*F. novicida zupT::*TN		(1)
NR-3063	*F. novicida znuA::*TN		(1)
MFN245	U112 FTN_0710::TN, FTN_1155::TN, FTN_1487::TN, FTN_1698::TN	U112 strain transposon insertions into restriction system genes.	(2)
BJM3141	*F. novicida* wild-type, pEDL56	*F. novicida* wild-type vector control	This study
BJM3148	*zur::*TN, pEDL56	*zur::*TN strain containing the vector control	This study
BJM3142	*zur::*TN, pEDL56::*zur*	Episomal complement of *zur::*TN	This study
BJM3145	*zupT*::TN, pEDL56	*zupT*::TN strain containing the vector control	This study
BJM3143	*zupT::*TN, pEDL56::*zupT*	Episomal complement of *zupT::*TN	This study
BJM3150	*znuA*::TN,	*znuA*::TN	This study

	pEDL56	containing the vector control	
F. tularensis Schu S4	Wild-type	*F. tularensis* subsp. *tularensis*	CDC
BJM1213	Wild-type, pEDL56::*znuA*		This study
BJM1214	Δ*znuA*, pEDL56::*znuA*		This study
BJM1230	Wild-type, pEDL56::*znuA*-HA		This study
pEDL56		TetR repressible expression vector	(3)
pBMBM363		U112 *zur* in pEDL56	This study
pBMBM361		U112 *zupT* in pEDL56	This study
pBMBM360		Schu S4 *znuA* in pEDL56	This study
pBMBM369		Schu S4 *znuA*+HA tag in pEDL56	This study
pGIR463		*sacB* suicide vector for genetic recombination	(4)
pBMBM353	pGIR463	Plasmid for genetic recombination of *znuA* gene	This study

1. Gallagher LA, Ramage E, Jacobs MA, Kaul R, Brittnacher M, Manoil C. 2007. A comprehensive transposon mutant library of *Francisella novicida*, a bioweapon surrogate. Proc Natl Acad Sci U S A 104:1009-14.
2. Gallagher LA, McKevitt M, Ramage ER, Manoil C. 2008. Genetic dissection of the *Francisella novicida* restriction barrier. J Bacteriol 190:7830-7.
3. LoVullo ED, Miller CN, Pavelka MS, Jr., Kawula TH. 2012. TetR-based gene regulation systems for *Francisella tularensis*. Appl Environ Microbiol 78:6883-9.
4. Ramakrishnan G, Meeker A, Dragulev B. 2008. fslE is necessary for siderophore-mediated iron acquisition in *Francisella tularensis* Schu S4. J Bacteriol 190:5353-61.

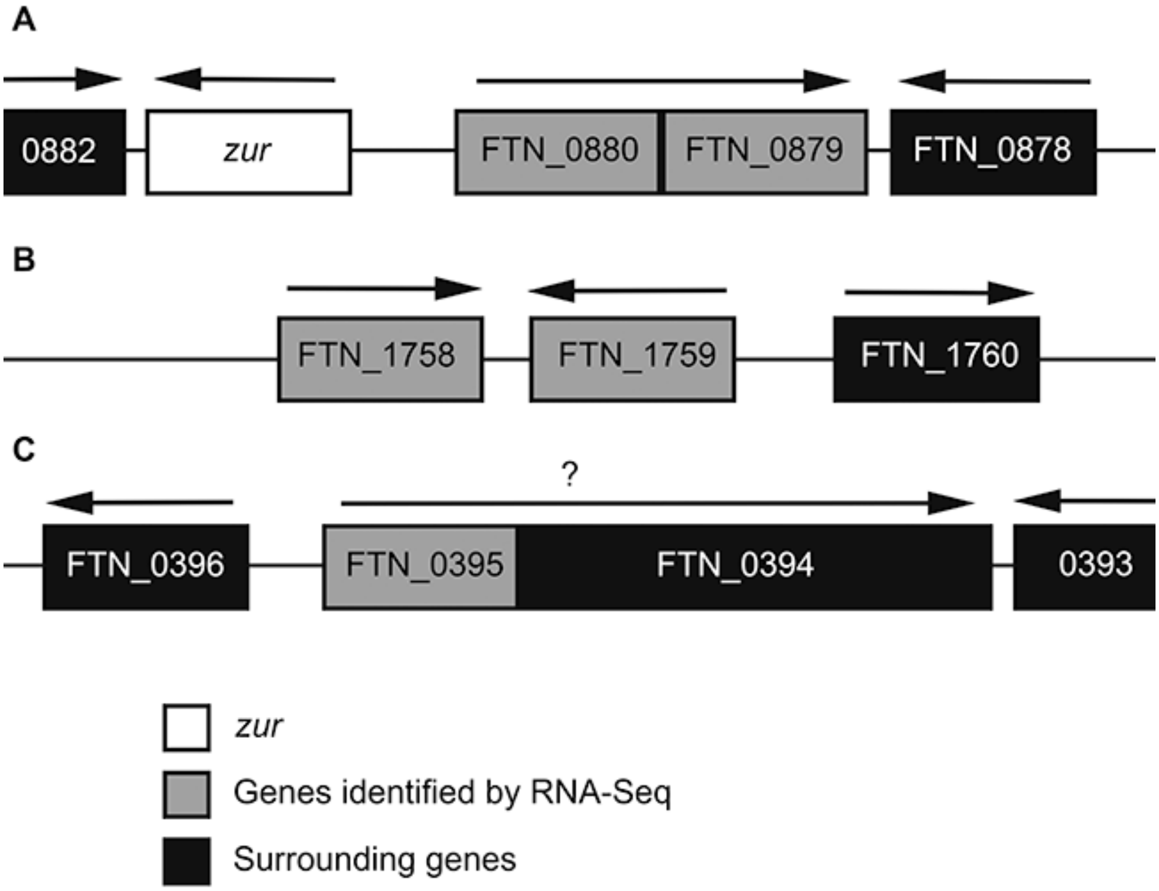

Supplemental Figure 1: Genomic organization of candidate genes.

Organization of genes identified by RNA-Seq with increased expression in an *F. novicida zur::*TN mutant. Identified genes are colored in grey. (A) FTN_0880 and FTN_0879 (*zupT*) are expressed as an operon. The *zur* gene (white) is located upstream of this operon and encoded on the opposite strand. (B) FTN_1758 and FTN_1759 are located near each other on opposite strands. (C) FTN_0395 is located directly upstream of FTN_0394, which is a predicted P-type heavy metal exporter.

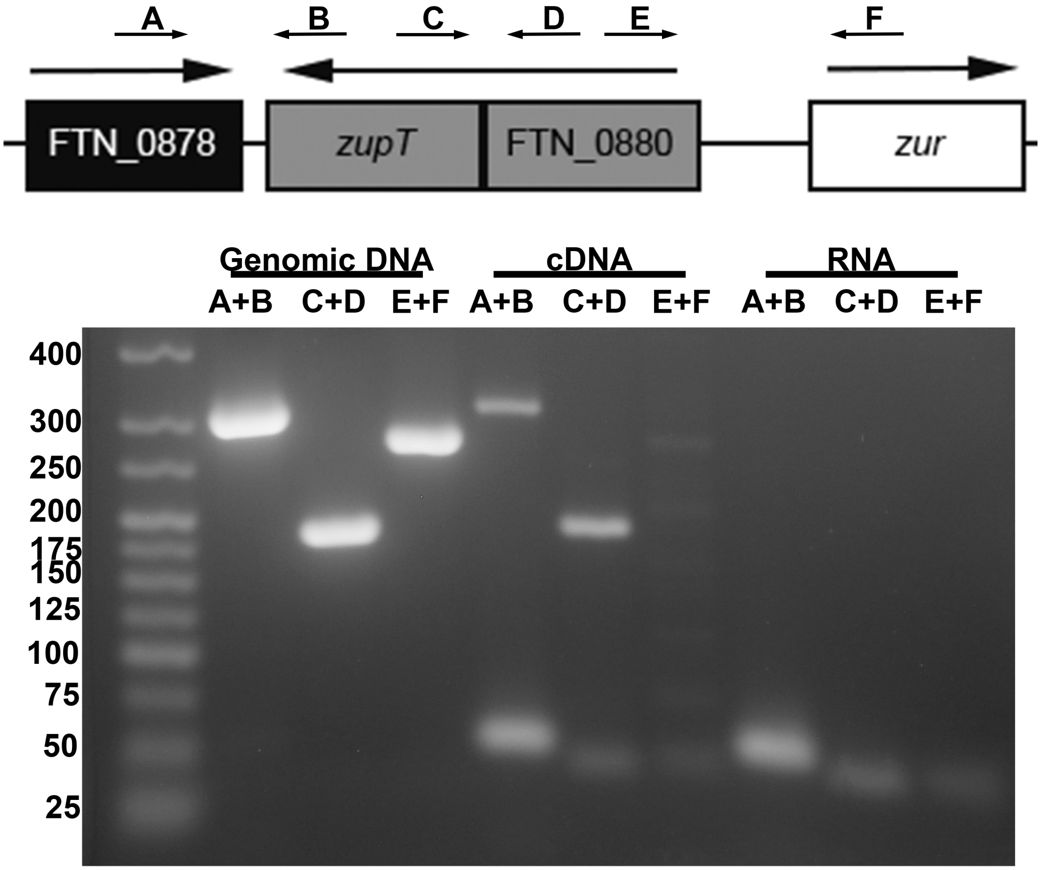

Supplemental Figure 2: *zupT* and FTN_0880 are transcribed as an operon. RT-PCR was performed to amplify the intergenic regions of indicated genes in cDNA generated from isolated U112 RNA. PCR products were separated on a 2% agarose gel. PCR with genomic DNA or RNA were used as positive and negative controls, respectively.

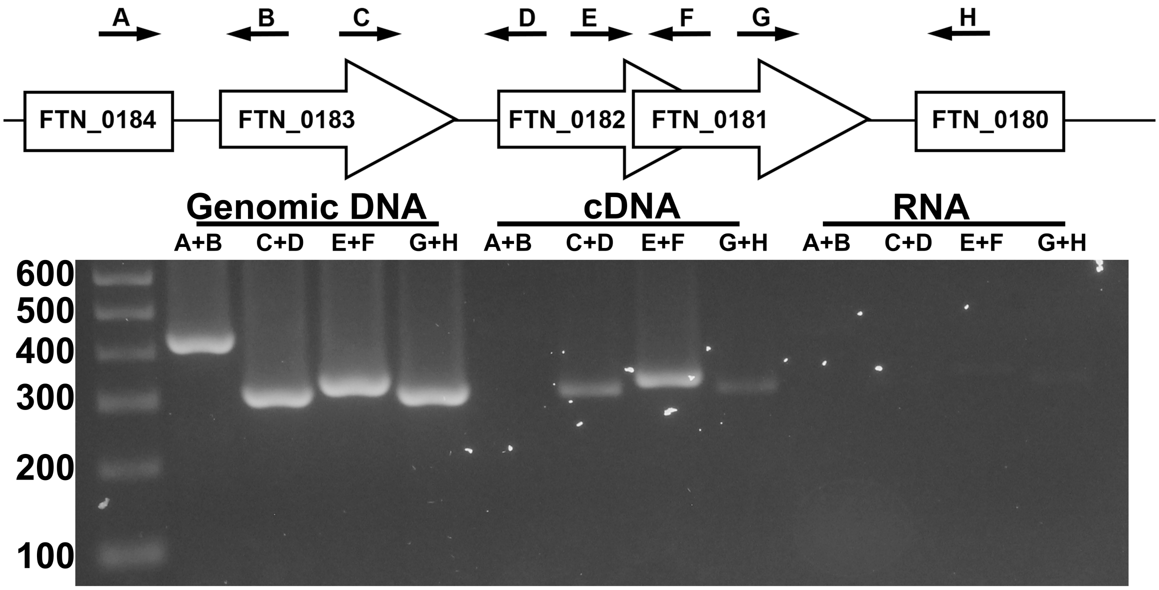

Supplemental Figure 3: FTN_0183-FTN_0181 are encoded as an operon.

RT-PCR was performed to amplify the intergenic regions of indicated genes in cDNA generated from isolated U112 RNA. PCR products were separated on a 2% agarose gel. PCR with genomic DNA or RNA were used as positive and negative controls, respectively.

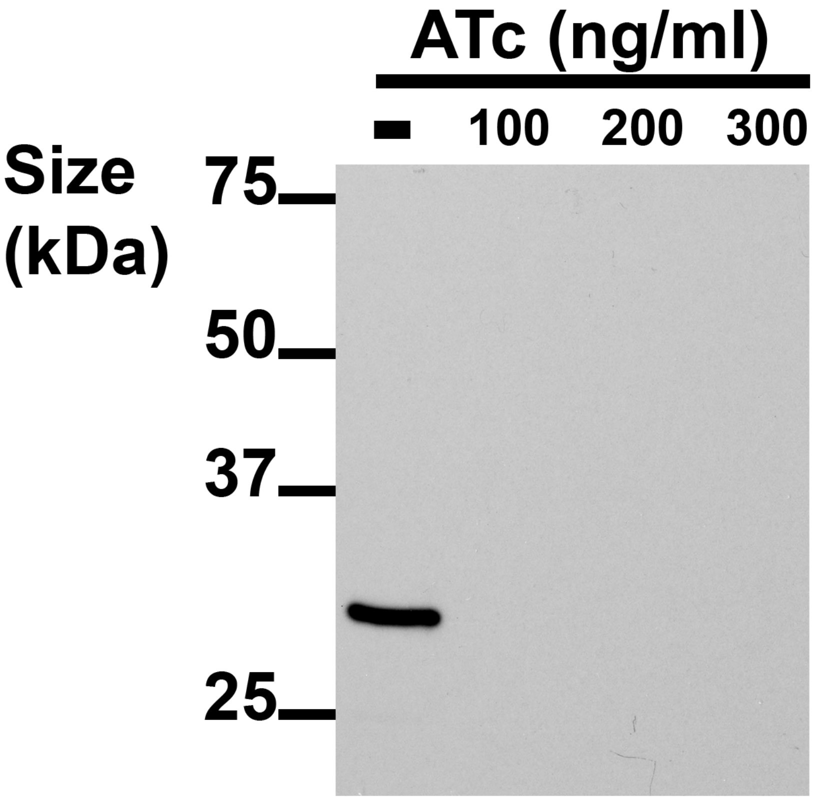

Supplemental Figure 4: Addition of ATc represses expression of ZnuA-HA protein. Blot of whole cell lysates obtained from wildtype p*znuA*-HA after 24 hours of growth in either untreated CDM or CDM supplemented with 100ng/ml, 200ng/ml, or 300ng/ml ATc. Whole cell lysates were loaded onto a polyacrylamide gel and transferred to a PVDF membrane. Nonspecific binding was blocked by incubating the blot overnight at 4°C in PBS-T with 5% milk. Blot was probed with 1:10000 mouse anti-HA followed by 1:5000 goat anti-mouse. Expected band size for ZnuA-HA is 33.7 kDa.
